# Dual-Pulsed Laser Ablation of Oyster Shell Producing Novel Thin Layers Deposed to *Saccharomyces cerevisiae*

**DOI:** 10.3390/polym15193953

**Published:** 2023-09-30

**Authors:** Georgiana Cocean, Alexandru Cocean, Silvia Garofalide, Vasile Pelin, Bogdanel Silvestru Munteanu, Daniela Angelica Pricop, Iuliana Motrescu, Dan Gheorghe Dimitriu, Iuliana Cocean, Silviu Gurlui

**Affiliations:** 1 Atmosphere Optics, Spectroscopy and Laser Laboratory (LOASL), Faculty of Physics, Alexandru Ioan Cuza University of Iasi, 11 Carol I Bld., 700506 Iasi, Romania; cocean.georgiana@yahoo.com (G.C.); silvia.garofalide90@gmail.com (S.G.); vasile.pelin@uaic.ro (V.P.); muntb@uaic.ro (B.S.M.); daniela.a.pricop@gmail.com (D.A.P.); dimitriu@uaic.ro (D.G.D.); 2 Rehabilitation Hospital Borsa, 1 Floare de Colt Street, 435200 Borsa, Romania; 3 Laboratory of Applied Meteorology and Climatology, Research Center with Integrated Techniques for Atmospheric Aerosol Investigation in Romania (RECENT AIR), Alexandru Ioan Cuza University of Iasi, A Building, Physics, 11 Carol I, 700506 Iasi, Romania; 4 Laboratory of Astronomy and Astrophysics, Research Center with Integrated Techniques for Atmospheric Aerosol Investigation in Romania (RECENT AIR), Alexandru Ioan Cuza University of Iasi, Astronomical Observatory, 11 Carol I, 700506 Iasi, Romania; 5 Sciences Department & Research Institute for Agriculture and Environment, Iasi University of Life Sciences, 3 Sadoveanu Alley, 700490 Iasi, Romania; imotrescu@uaiasi.ro

**Keywords:** dual-pulsed laser induced chitin deacetylation, chitosan encapsulated biological cells, miniaturizing pulsed laser deposition systems, transdermal drug delivery systems

## Abstract

Dual-pulsed (DPL) laser deposition using oyster shells as targets was studied in order to find out if this method can replace the use of high-power pulsed lasers. Aspects related to changes in the morphological structure of the thin layer but also to the chemical composition of the obtained thin layer were analyzed and compared with the target as well as with the thin layers obtained with a higher power pulsed laser in a single-pulsed (SPL) regime. Orthorhombic structures were noticed with Scanning Electron Microscopy for the thin film obtained in DPL mode compared to the irregular particles obtained in SPL mode. The deacetylation process during ablation was evidenced by Fourier Transform Infrared spectroscopy, resulting in chitosan-based thin films. The effect of the obtained thin films of chitosan on the cells of baker’s yeast (*Saccharomyces cerevisiae*) was studied. Restoration of the yeast paste into initial yeast was noticed mainly when the hemp fabric was used as support for the coating with yeas which was after that coated with chitosan thin film produced by DPL method.

## 1. Introduction

High-power pulsed laser irradiation of oyster shell was proved to produce chitin deacetylation and resulted in the deposition of thin films of chitosan and the new LICD method (laser-induced chitin-to-chitosan deacetylation) was reported [1]. The novel method of extracting biopolymers (such as chitosan, alpha-keratin, curcuminoids, and hemp constituent biopolymers) by laser ablation directly from raw biomaterials (oyster shell, horn, wool fiber, turmeric, and hemp stalk) does not require chemical processing or other treatments, relying only on the processes induced by pulsed laser radiation [1,2,3,4]. Previous to the above-cited studies, laser procedures had only been used on already extracted or synthesized polymers. Also, according to our knowledge, until now there have been no other studies involving the production of polymers by applying laser ablation directly on raw biological materials.

In this context, the aim of the study presented herein is to investigate the effects of dual-pulsed laser systems on the biocomposite represented by oyster shells. The dual-pulsed laser (DPL) system is a new concept on how the high-power laser could be supplemented either because of the limit to which such laser systems can be built in general or because of the limitation due to the need to incorporate them into miniaturized devices. This study aims to identify changes in the morphological and chemical structure of the materials consisting of polymers during ablation and deposition in vacuum conditions when for irradiation, a dual-pulsed laser system (DPL) is used. Also, the effects of the DPL mode-produced chitosan thin film on bio-cells will be investigated.

Chitosan as a linear polymer extracted from crustaceans, clams, and insects by deacetylation of chitin has been intensely studied due to its benefits in medicine, food preservation, and other domains. Chitin deacetylation using chemical procedures is one of the most used methods to produce chitosan and the technique has been continuously improved in order to achieve a higher deacetylation degree of chitin [5,6,7,8,9,10,11,12]. Enzymatic deacetylation is another well-known procedure to obtain chitosan from chitin. Using chitinase, chitin deacetylation can be performed on different shells and the process can be conducted further to chitosan modification when chitosanase is used [13,14,15,16]. The procedures for isolating and purifying the enzymes used in the degradation of polysaccharides, including enzymatic deacetylation of chitin, are quite expensive and limit their accessibility [17,18]. Bacterial and fungal degradation of chitin has also been performed for over 20 years and its mechanism studied, providing information on the biochemical chitin deacetylation specificity involving chitinolytic interactions depending on the habitat structure and environmental parameters such as temperature [19].

Similar to cellulose, chitin has an ordered fiber structure, with intra and intermolecular hydrogen bonds, a high degree of crystallinity and polymorphism. The rigid chitin chain is insoluble in common solvents (water or alcohol). Chitin activation is usually achieved by swelling or partial degradation and requires the use of an excess of aggressive chemical reagents. Non-chemical alternatives for chitin activation were studied and the process of plastic deformation under pressure and shear was found as suitable for achieving a high degree of deacetylation and fewer crystalline regions [20]. Also, a biocompatible deacetylation alternative was proposed with the use of eutectic solvents (DES), and mixtures of pure compounds (a hydrogen bond acceptor and a hydrogen bond donor) [21].

In order to meet functional application parameters, fabrication methods and treatments of chitin and chitosan have been investigated. Modified physicochemical properties of chitosan by mechanochemical disorder and amorphization, plasma treatment and copolymerization were reported [22].

Chitosan is known for its biodegradability, biocompatibility and mucoadhesive properties. The crystalline structure of chitosan is deeply dependent on its deacetylation process and on its polymorphic form of chitin. Studies related to the cleaning activity of superoxide radicals (from the body) with the help of α and β chitosan nanoparticles have shown good efficiency in relation to the dose. Chitin presents three polymorphic forms of chain arrangement in the crystalline region. They can be arranged antiparallel as α-chitin, parallel as β-chitin or a combination of both as γ-chitin. In α-chitin, other hydrogen bonds and van der Waals forces strongly bind the sheets together, with the distinct feature expressed by the appearance of two distinct conformations of the primary hydroxyl groups [23]. In α-Chitin, hydrogen bonds and van der Waals forces strongly bind the sheets together, with the appearance of two distinct conformations of the primary hydroxyl groups. In the case of β-chitin, the swelling and penetration of polar molecules do not cause a major distortion of the organization of the sheet. On the other hand, if the acid is removed, alpha-chitin crystallinity is restored along with the destruction of beta-chitin crystals and the formation of new α-chitin crystals. Okuyama [24] and Ogawa [25] proposed the two crystallization forms of chitosan which are currently known: hydrated orthorhombic allomorph and anhydrous allomorph, respectively. The hydroxyl and amino groups contained in the backbone of the polymer condition the antioxidant activity of chitosan by eliminating anionic radicals, such as superoxide and hydroxyl radicals. This fact, however, depends on a series of properties—such as the degree of deacetylation (% DD), the molecular weight (MW), the source of origin of chitin, and the reaction conditions. Chitosan nanoparticles obtained due to electrostatic interactions between amino groups and polyanionic crosslinking agents change their functional properties, as a result of the crystalline structural differences between α- and β-chitosan [22]. It was observed that with the increase in chitosan concentrations, morphological and ultrastructural changes occur inside the fungal cell wall as a result of the increase in chitin deposits [26].

Several reports have noted the modulation of the assembly mode of chitosan chains with the electrical signals [27]. Composites and nanocomposites based on chitosan have been studied and produced for electronic components [28,29] and sensors [30]. Conducting polymers are of interest also in tissue engineering [31]. Artifact preservation is also a field of application of chitosan in fungicide treatment [25]. Polysaccharides’ protective properties against metal corrosion have also been demonstrated [32].

## 2. Materials and Methods

**Figure 1:**
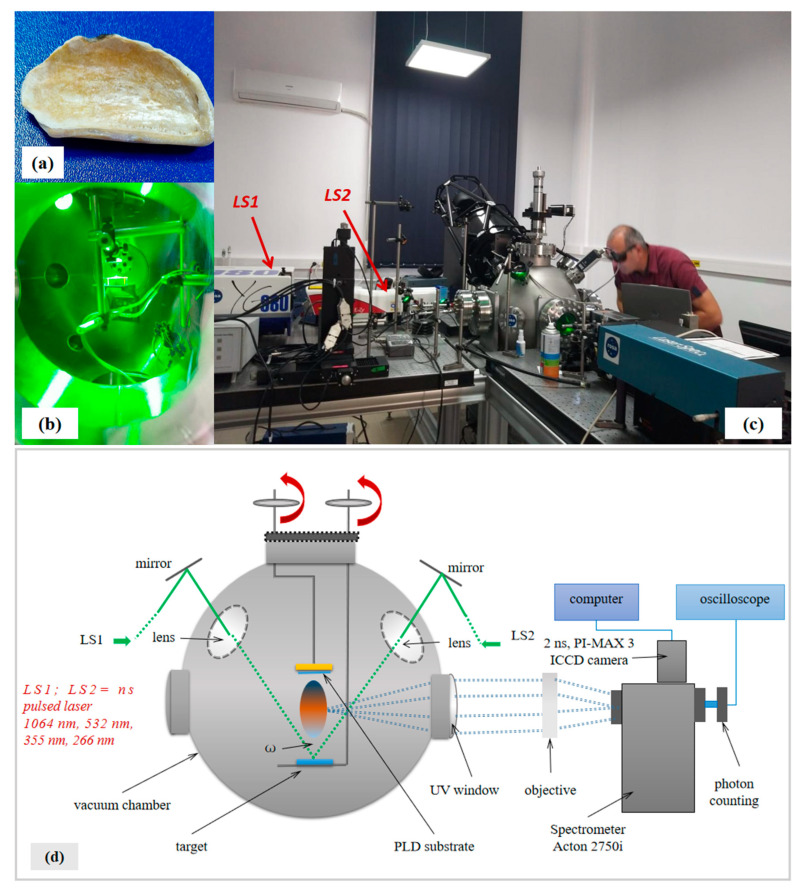
Images of oyster shell target (**a**), deposition chamber during PLD performed with dual-pulsed lasers system on oyster shell target (**b**), installation during work (**c**) and schematic representation of PLD installation with dual-pulsed lasers system (**d**).

The material used as a target for pulsed laser deposition (PLD) was a piece of oyster shell (Figure 1a) from the Black Sea, Golden Sands, Varna, Bulgaria. The oyster shell was washed and dried without any other preparation and/or treatments. Oyster shells are natural biocomposites consisting of a matrix of calcium carbonate with the main component chitin as a dispersed phase which also includes different minerals and organic compounds [1].

Glass slab and hemp fabric (same as in previous experiments reported by [1,2,3,4,33]) were used as supports for thin film deposition. Dry baker’s yeast (*Saccharomyces cerevisiae*) acquired from commerce (produced by Dr. August Oetker Nahrungsmittel K. G., 33547 Bielefeld, Germany) was softened with water enough to form a paste with which both a glass and a hemp fabric substrate were coated. The yeast paste was placed on one edge of the material and then spread with a spatula over the entire surface. Thin layers were also deposited on the coated substrates using the same shell as for the depositions on the uncoated glass slab and the uncoated hemp fabric.

The experimental work was conducted in the deposition chamber (Figure 1b) of the installation as shown in Figure 1c,d using one laser (LS1) or both lasers (LS1 and LS2) for single and dual-pulsed lasers operating regime (SPL and DPL, respectively). The pressure in the deposition chamber was 2 × 10^−2^ Torr.

For this experiment, the working parameters of the two laser systems acquired from Quantel, Les Ulis, France are Q-switched Nd:YAG lasers were set to 532 nm beam wavelength, 10 nm pulse width and 10 Hz repetition rate.

**Figure 2:**
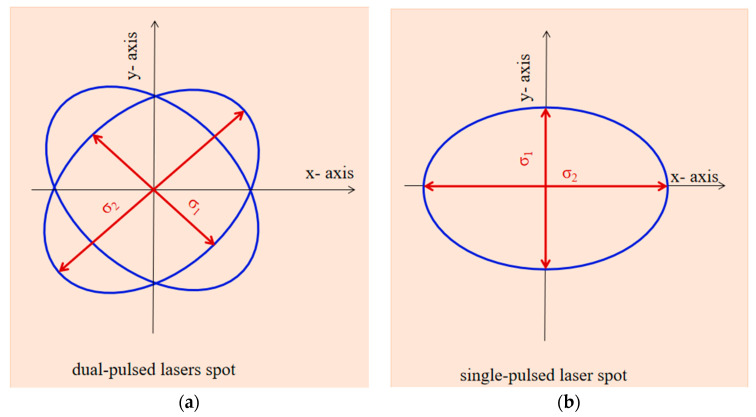
Dual-pulsed laser spot (**a**) and single-pulsed laser spot (**b**).

In the single-pulsed laser regime, the laser beam energy used was 60 mJ/pulse. The laser beam radius was 325 μm resulting in an elliptical spot shape of σ_x_ = 650 μm and σy = 919 μm standard deviations (Figure 2a,b) at the incidence with the target surface under an angle α = 45°.

In the dual-pulsed laser regime, the target was simultaneously irradiated with the two laser systems, each of them operating at 30 mJ/pulse energy, and the angle ω between the two laser beams was 45°, as illustrated in Figure 1d. The laser beam radius of 325 μm results in an elliptical spot shape of σ_x_ = 650 μm and σy = 919 μm standard deviations (Figure 2a,b) at incidence with the target surface under an angle α = 45°.

The deposition time for producing each thin film was set to 30 min and the support for deposition was placed to 3 cm distance from the target. The sizes of the glass slab used as deposition support measure 1 inch in width by 3 inches in length.

The experimental study was assisted and completed by a numerical simulation in COMSOL v5.6 software (COMSOL AB, Stockholm, Sweden) to estimate thermal conditions developed in a chitin–calcium carbonate composite material under the irradiation with two laser beams of 30 mJ/pulse each (dual-pulsed lasers 2 × 30 mJ/pulse) compared to single-pulsed laser of 60 mJ/pulse and evaluate plasma threshold. The parameters and conditions used in the simulation were in accordance with the experimental ones.

The theoretical and numerical models implemented are as previously reported [1,4,34,35,36]. For the dual-pulsed laser regime, the density of power, I0* Wm2 in the heat source equation (*Q*) in the “Variables” section of the “Heat Transfer in Solids” COMSOL module was introduced as the result of the composition of the power density vectors related to each laser radiation. Under these conditions, the scalar value of the resulting density of power, I0* Wm2, is given by the equation:

$$I_{0}^{*}=I_{0}\cdot \sqrt{2\cdot \cos\omega },$$

where $I_{0}=\frac{E\;(J)}{\tau (s)\cdot A(m^{2})}$ is the peak density of power of each of the lasers used in the dual regime and it is calculated based on the laser beam energy *E* (*J*), pulse width *τ* (*s*) and spot area *A*(*m*^2^). The laser power absorbed by the material as a heat source, *Q*, is now given by the equation:

$$Q(x,y,z,t)=I_{0}\cdot \sqrt{2\cdot \cos\omega }\cdot \left(1-\frac{{\left(n\left(T\right)-1\right)}^{2}+k^{2}(T)}{{\left(n\left(T\right)+1\right)}^{2}+k^{2}(T)}\right)\cdot \frac{4\pi k^{2}(T)}{\lambda }\cdot e^{-{\frac{\left(x-x_{0}\right)}{2\cdot \sigma_{x}^{2}}}^{2}-\frac{{\left(y-y_{0}\right)}^{2}}{2\cdot \sigma_{y}^{2}}}\cdot e^{-3.5\cdot {\left(\frac{t-\tau }{\tau }\right)}^{2}}\cdot e^{-\frac{4\pi k^{2}(T)}{\lambda }\cdot \left|z\right|}$$

where *λ* is the wavelength, *n* is the refractive index and *k* is the extinction coefficient. A parametric sweep on the angle between the two laser beams, *ω* = (0°, 15°, 20°, 30°, 45°, 60°, 90°), was introduced in the simulation setup. For parameters and constants of the materials (calcium carbonate and chitin), the COMSOL library data were used and completed by online resources [1,34,35,37].

Fourier Transform Infrared Spectroscopy (FTIR) was performed with the FT-IR Spectrometer Bomem MB154S spectrometer at an instrumental resolution of 4 cm^−1^ (Bomem, ABB group, Québec, QC, Canada) on the thin films obtained in the DPL regime compared to the target in order to evaluate deacetylation process having as reference the results previously reported [1]. The pallets with the material to be analyzed were obtained by mixing with KBr the powder collected from the samples by the procedures already reported [1]. The FTIR analysis was assisted by numerical simulation in Gaussian 6 software of the IR spectra of chitin, calcium carbonate and chitosan. The morphological structure and the elemental composition were evaluated with the SEM-EDX technique (Scanning Electron Microscope coupled with Energy Dispersive X-ray, Vega Tescan LMH II, Brno, Cehia). Using Toup Tek Toup View software, version x64, 4.11.19728.20211022, the particle sizes were evaluated on the SEM images. Thin layer thickness and roughness were measured with a DektakXT stylus profilometer, manufactured by Bruker Billerica, MA, USA.

## 3. Results and Discussions

### 3.1. Experiment Optimization with Numerical Analysis in COMSOL V5.6

**Figure 3:**
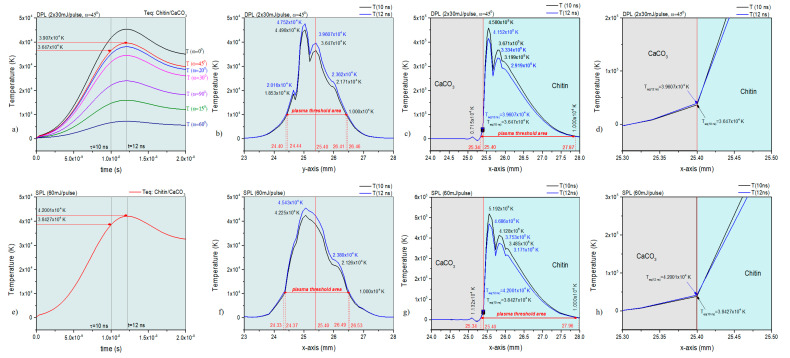
Plasma threshold illustrated in the 1D diagrams generated based on the COMSOL simulation: T(t) diagram in spot center in DPL regime (**a**); T(y) diagram, DPL regime, 10 ns and 12 ns (**b**); T(x), DPL regime, 10 ns and 12 ns (**c**,**d**); T(t) diagram in spot center in SPL regime (**e**); T(y) diagram, SPL regime, 10 ns and 12 ns (**f**); T(x), SPL regime, 10 ns and 12 ns (**g**,**h**).

The COMSOL simulation of the DPL and SPL irradiation of the two main components in the biocomposite material of the oyster shell, calcium carbonate (CaCO_3_) and chitin provided information on the plasma threshold (Figure 3a–h). The temperature variation in time in the spot center in Figure 3a shows for the DPL regime the highest peak temperature for an angle ω = 45° between the two laser beams, as expected, ω = 0° being irrelevant in this case.

The existence time of the plasma threshold extends beyond 20 ns. The same effect in time for the plasma threshold is also observed in the SPL regime (Figure 3e). The thermal effects induced by the laser irradiation and dispersed in the material by heat transfer in solids in DPL and SPL regime analyzed for the two main components denote high temperatures compatible with plasma threshold (higher than 10^4^ K order) on areas of 2.06 mm along *y*-axis and 2.53 mm along *x*-axis in DPL regime compared to the areas of 2.20 mm along *y*-axis and 2.62 mm along *x*-axis in SPL regime.

The *y*-axis is assigned to a homogeneous mixture of the two components while the *x*-axis is assigned to a larger dispersion of the two components in the mixture, each component exhibiting more of its optical and heating properties in the latter situation. Important for the study presented herein is that the plasma conditions obtained in the DPL regime with *ω* = 45° are similar to those in the SPL regime.

**Figure 4:**
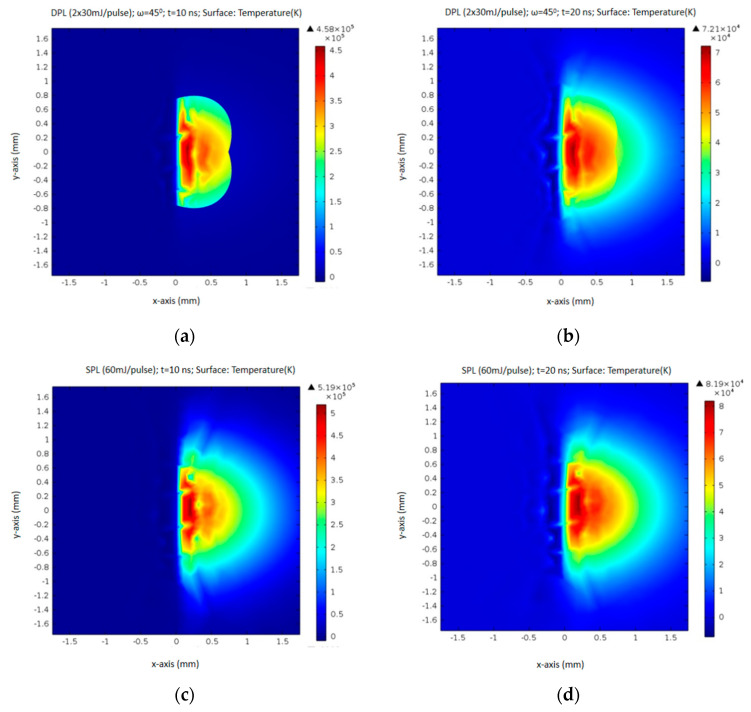
Compared thermal effects on the ablated surface in DPL regime 10 ns (**a**) and 20 ns (**b**) after pulsed laser ignition and SPL regime 10 ns (**c**) and 20 ns (**d**) after pulsed laser ignition.

By further analyzing the simulation results through 2D diagrams for the irradiated surfaces of the two main components, significant differences were found regarding the appearance of the thermal spot (Figure 4c,d). Thus, the thermal spot in the SPL regime at 10 ns (Figure 4a) is about half as wide as that in the SPL regime at the same time of 10 ns (Figure 4c). After 20 ns, the two thermal spots, both in DPL and SPL regimes, become close in extent (Figure 4b,d). Observing the similar extent of the surface of the spots in SPL mode at 10 ns (Figure 4c) and SPL at 20 ns (Figure 4d), it can be assessed that the simulation shows that the thermal diffusion in the material generated by the source in DPL mode starts later than that in SPL mode. In the latter, the thermal diffusion is already effective at 10 ns, while in the DPL regime, the effect of thermal diffusion is much reduced at 10 ns. Important for our study is that, at 10 ns, when the peak temperatures are also higher by an order of magnitude (10^5^ K versus 10^4^ K after 20 ns), they are also more homogeneous on the thermal surface, which is actually the ablated surface. As was presented in the previous work [1], the calcium carbonate component does not absorb laser radiation, while the chitin component highly absorbs, the laser radiation and heating of the calcium carbonate through heat transfer from the chitin will lead to its decomposition to a large extent, so that some carbonate could be also entrained in the ablation process.

The influence of these differences will be analyzed in the results obtained experimentally for the thin layers.

### 3.2. Morphological and Chemical Analysis of the Thin Film Obtained in Dual-Pulsed Lasers Regim

#### 3.2.1. SEM–EDX Morphological and Elemental Composition Analyses

**Figure 5:**
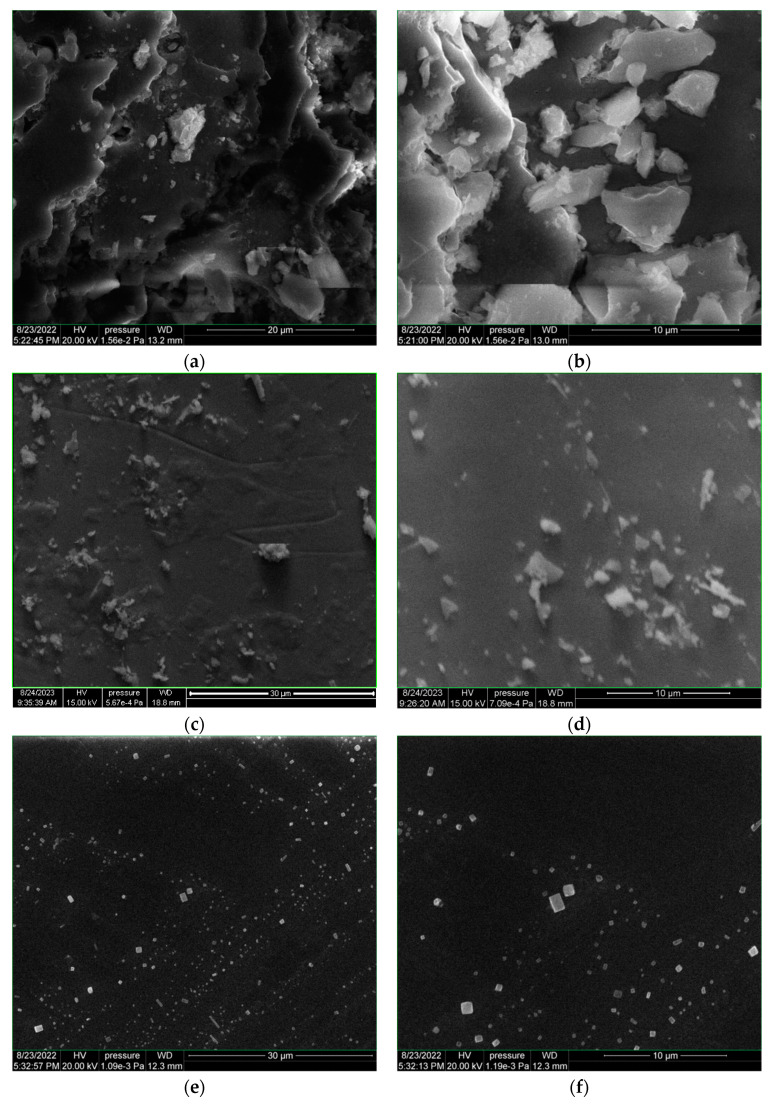

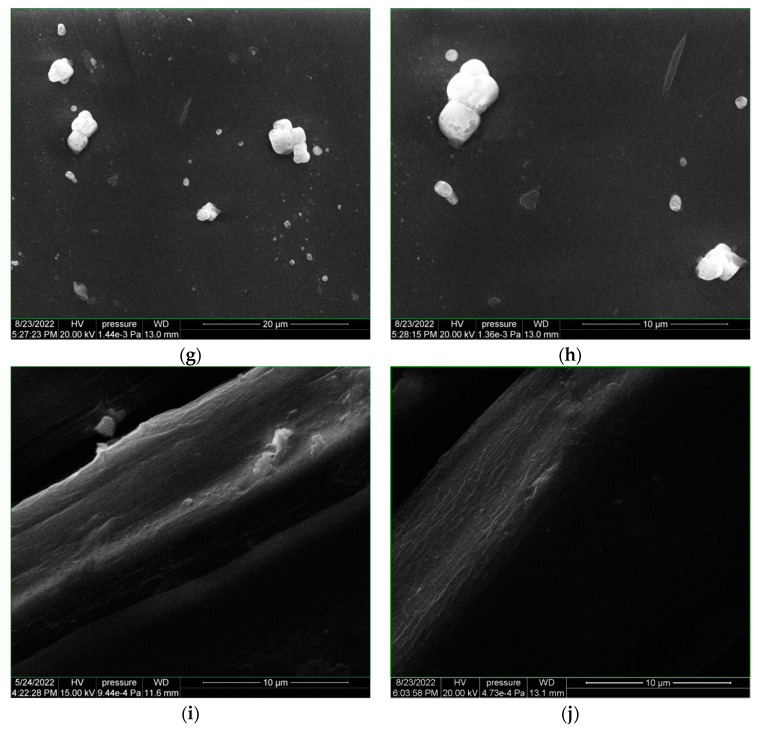
SEM images of the oyster shell target (**a**,**b**); PLD thin films obtained with single-pulsed laser of 60 mJ/pulse on glass slab (**c**,**d**); PLD thin films obtained with dual-pulsed lasers 2 × 30 mJ/pulse on glass slab (**e**–**h**); hemp fabric (**i**); PLD thin film obtained with dual-pulsed lasers 2 × 30 mJ/pulse on hemp fabric as support (**j**).

**Figure 6:**
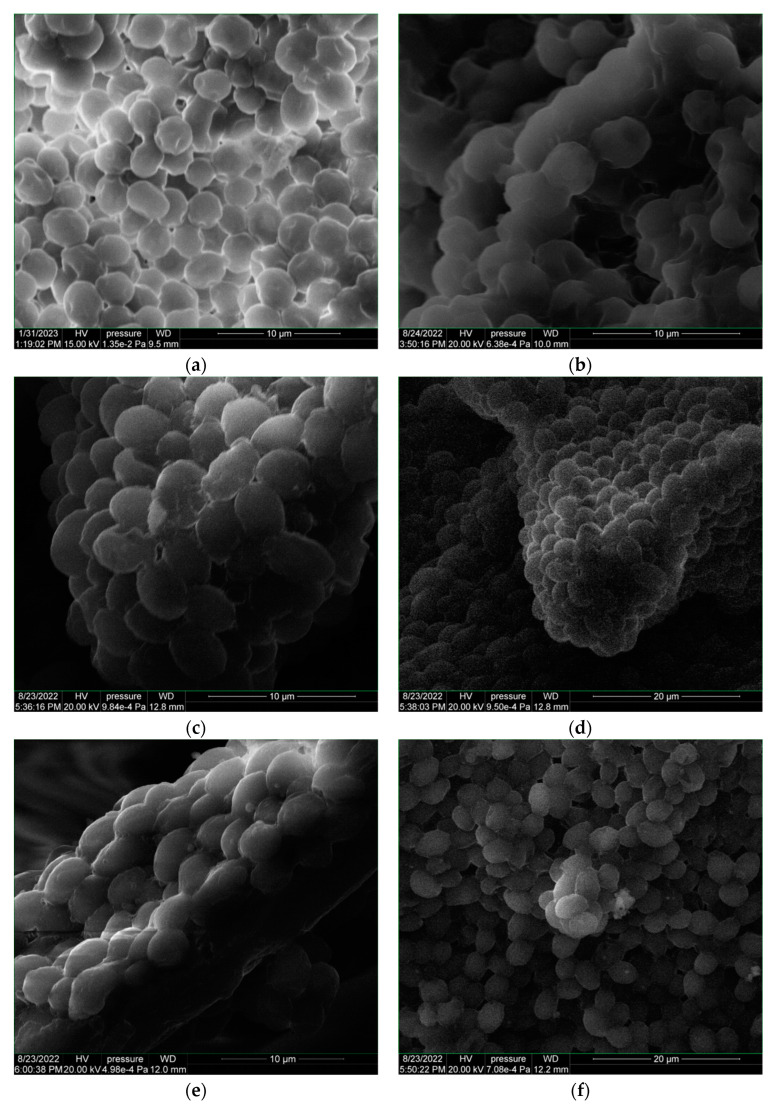
SEM images of dry yeast (**a**); yeast coating support (**b**); OS-Y-glass (**c**,**d**); OS-Y-hemp (**e**,**f**).

Scanning Electron Microscope coupled with Energy Dispersive X-ray (SEM-EDS) was performed on the oyster shell target (OS-target) and the thin films obtained on glass support in DPL and SPL regime (Figure 5a–h). Thin films deposited in DPL mode on hemp fabric (Figure 5i,j) and on yeast coatings were also the subject of SEM-EDX analysis (Figure 6a–f).

The main observation is the totally different shape of the structures of the particles deposited in the DPL regime compared to the SPL regime, despite the very similar conditions in terms of total energy per pulse applied on the target.

In the SPL mode, with 60 mJ/pulse laser beam energy, the particles preserved the morphological structure with the one observed for the target constituents, only of smaller size (Figure 5a,d). In this sense, from structures with sizes between 10 μm and 7.5 μm and even larger than, those of 0.5 μm measured on the target, structures with sizes between 2.85 μm and 0.3 μm were obtained in the SPL regime on the thin film.

On the other hand, in the DPL mode (Figure 5 e,f), two types of structures were obtained, both totally different in shape from those observed on the target and on the thin film from the deposition in the SPL mode. The predominant structures from the DPL deposition (2 × 30 mJ/pulse) are the orthorhombic ones, of various sizes, the largest with a size of 1.2 μm, the vast majority having a size smaller than 1 μm, some with dimensions of the order of 200 nm. It is also worth noting the arrangement of these orthorhombic particles in a linear V-shaped pattern. This V-shaped pattern is similar to the trajectory—also in V—of the two laser beams. The phenomenon requires a more in-depth study in order to understand both the mechanism of the formation of orthorhombic structures and that of inducing the V arrangement of the respective particles.

The phenomenon is all the more interesting since on the same DPL deposition, granular structures (close to ovoid shape) were also found, with sizes of 2.5 um, 2 um and 1 um, forming agglomerations of 6.25 um/3.75 um. However, these later structures are rare on the thin film surface. After the deposition obtained in the DPL regime on the hemp fabric, a smooth appearance effect on the hemp fiber compared to the initial fiber was observed (Figure 5i,j).

For the deposition in DPL mode on the glass and hemp support coated with yeast paste, namely OS-Y-glass and OS-Y hemp, respectively, a restructuring of the initial cells of the dry yeast (Y) is observed compared to that of the dried yeast paste (Y-support) without the deposition of the thin layer from oyster shell (Figure 6a–f).

The elemental composition of the thin film is influenced by the substrate as can be seen in Table 1 when comparing the data obtained for the thin film and those of the different substrates used. However, a significant decrease in the calcium content of the thin films obtained in DPL mode compared to the oyster shell target can be observed, as anticipated by the simulation in COMSOL. The calcium atomic percentage (number of atoms of a species out of 100 atoms of all detected species) mitigates from 8.39% in the oyster shell to average 0.23% in OS-glass, 0.06% in OS-hemp, 0.13 in OSY-glass and OSY-hemp.

#### 3.2.2. Fourier Transform Infrared Spectroscopy (FTIR)

The FTIR analysis of the thin films obtained by deposition in the DPL regime was carried out by comparison with the materials used when they were produced.

**Figure 7:**
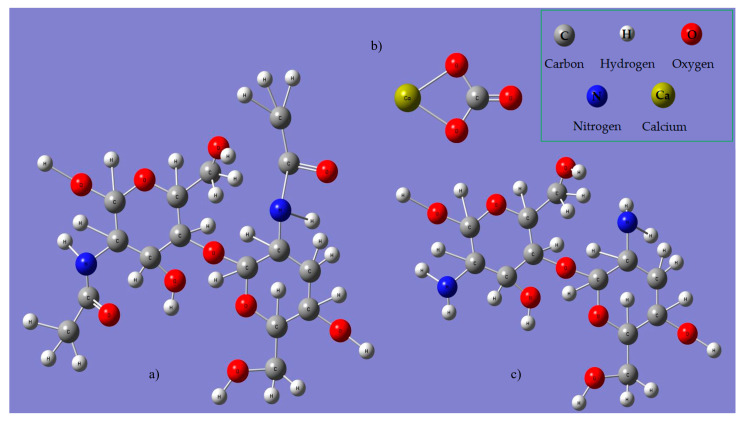

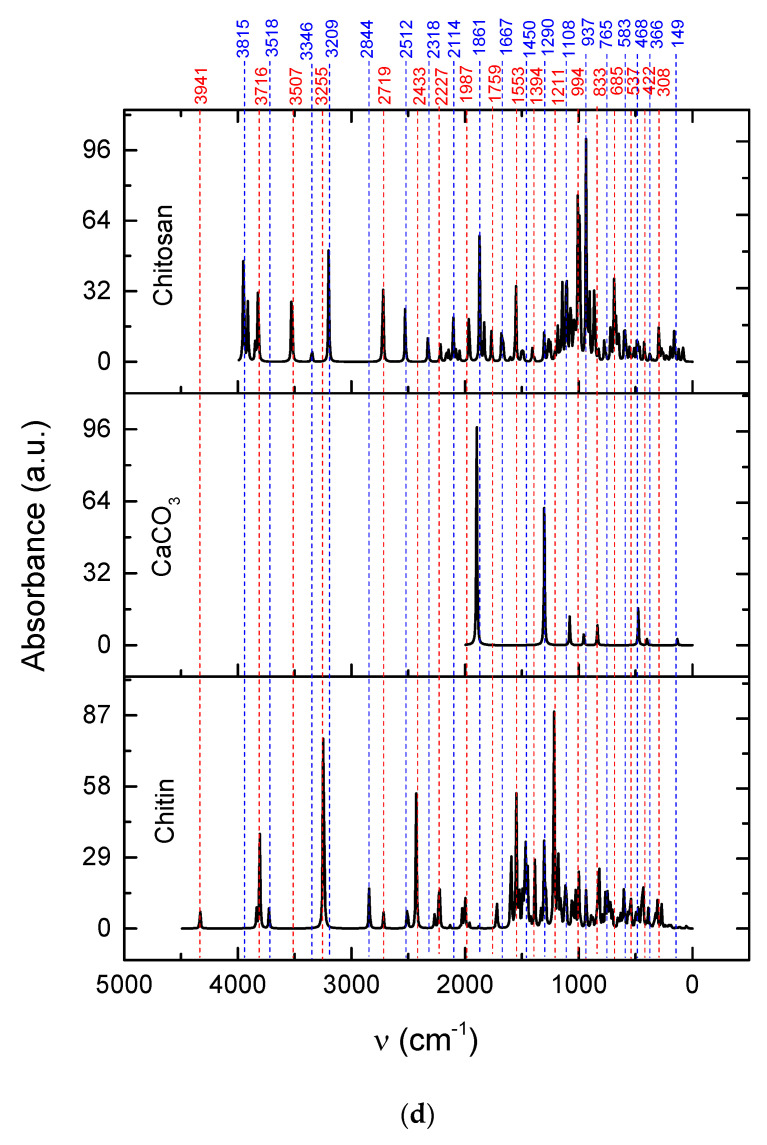
Simulation in Gaussian 6 software of the structural formulae of chitin, β-(1,4)-N-acetylglucosamine (**a**); calcium carbonate, Ca CO_3_ (**b**); chitosan, β-(1,4)-D-glucosamine (**c**); and of their IR spectra (**d**).

**Figure 8:**
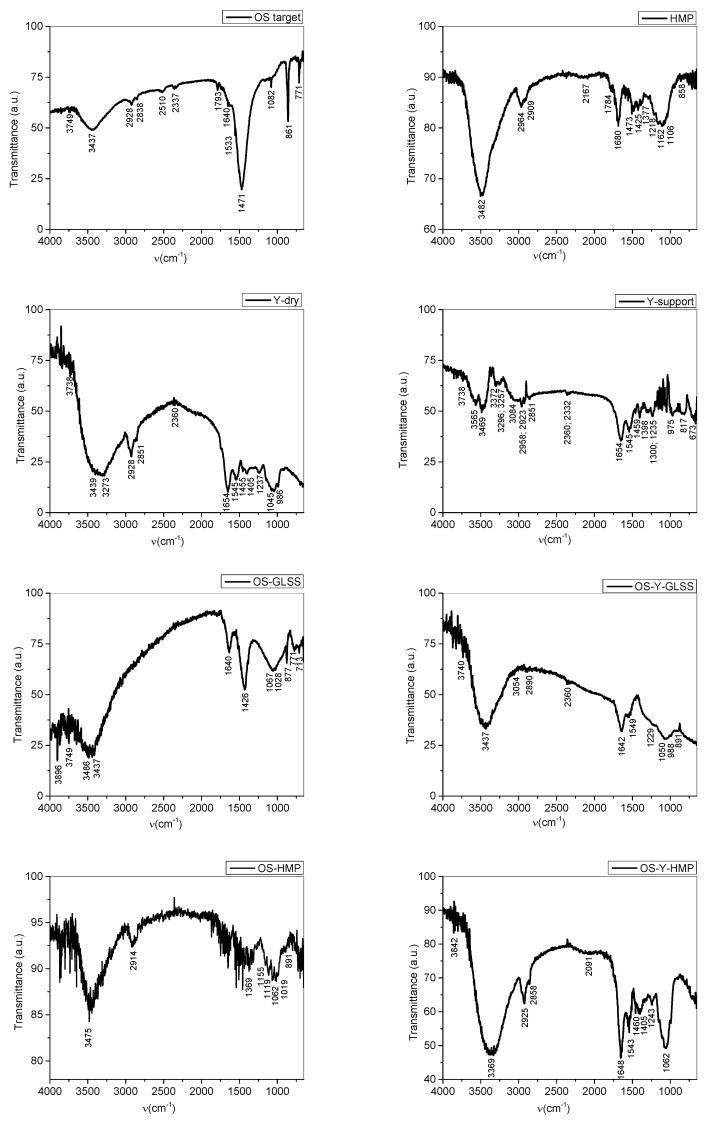
Compared FTIR spectra of oyster shell target (OS target); hemp fabric (HMP); dry yeast (Y-dry); yeast coating material (Y-support); PLD oyster shell film deposition on glass slab (OS-GLSS); PLD oyster shell film deposition on the yeast coating the glass slab (OS-Y-GLSS); PLD oyster shell film deposition on hemp fabric support (OS-HMP); and PLD oyster shell film deposition on the yeast coating the hemp fabric (OS-Y-HMP).

The structural formulae of chitin, calcium carbonate and chitosan were generated using Gaussian 6 software and their molecule’s IR spectra were simulated (Figure 7a–d). The results obtained in the Gaussian 6 simulation were used to assist the interpretation of the functional group vibrations obtained in the FTIR spectra presented in Figure 8. It must be mentioned that the simulation only provides information on the specific vibrations of the molecular functional groups and does not take into account the specific intra- and inter-molecular interactions such as hydrogen bonds and van der Waals interactions, nor tautomery due to double bonds.

The measured IR vibrations using FTIR spectroscopy of the functional groups of the chemical compounds contained in the analyzed materials are presented in Table 2 together with the interpretation based on the literature and the vibrations in chitin, calcium carbonate and chitosan IR spectra obtained in the Gaussian 6 simulation.

Chitin deacetylation during deposition in DPL regime is observed in the OS-GLSS spectrum where there is only one peak in the 1800–1700 cm^−1^ range of C=O functional groups. The vibration at 1640 cm^−1^ is assigned to NH_2_ group resulting in the chitosan molecule after C=O group was removed in the deacetylation induced during DPL ablation of chitin. Also, the strong peak at 1471 cm^−1^ assigned in literature [39] to carbonate ions, is diminished in intensity compared to the OS target. This vibration in the OS-GLSS spectrum is assigned to OH bendings which overlap on the carbonate vibration. In the same range, lattice vibrations are assigned. In the thin film, it indicates the crystalline structure of chitosan [1]. The crystalline structure and hard particles which were not crushed into powder during mortaring for the pellets produced for FTIR analysis were also indicated by the basic line shape of the Mie scattering effect. The missing peak in the 1800–1700 cm^−1^ range of C=O functional group in amides is also noticed for the spectra of the thin films deposited on the other supports (OS-HMP, OS-Y-GLSS, OS-Y-HMP).

The increased sorption of water due to the chitosan thin film is also observed by the “noise-like” effect noticed mostly in the 4000–3500 cm^−1^ range on the OS-GLSS, OS-Y-GLSS, and OS-Y-HMP. As previously reported [1], for the high-power pulsed laser deposition of chitosan thin films from oyster shell targets on hemp fabric, the sorption of water is highly increased, as the OS-HMP spectrum in Figure 8 proves by the very noisy spectrum line. Gas sorption could be also assigned to the noticed shape line of the spectrum.

Important to notice is the effect on the yeast (Y-support) of the thin film of chitosan produced by DPL deposition mode from oyster shell. Comparing the Y-support spectrum to the Y-dry spectrum, a very significant change is noticed which denotes the chemical changes associated with the already noticed morphological changes in Figure 6b versus Figure 6a. The split of the 3565–3469 cm^−1^ peak in two in the Y-support spectrum denotes NH_2_ group formation in the yeast cell molecule which denotes that C=O was eliminated as CO_2_ in the fermentation process.

Depositing the chitosan thin film, yeast cells were either sealed and the fermentation process was interrupted, and the cells were “preserved”, yeast cells were either sealed and the fermentation process was interrupted, or other cellular “restructuring” or “healing” processes took place through the interaction between the molecules of the particles that make up the thin film and the cells of yeast. To explain the phenomenon, in-depth microbiology studies are needed, including on other biological cells. In the case of using the hemp substrate covered with yeast paste for the deposition of chitosan by the DPL method, there is even a restoration of the molecular structure of the dry yeast, and the SEM images (Figure 6e,f versus Figure 6a) are in agreement with the results of the FTIR spectrum (Figure 8 OS-Y-HMP versus Y-dry).

#### 3.2.3. Profilometry of the DPL Thin Film

The thin film thickness analysis using the profilometry method involves two components: the step (the difference of height between the support and the thin film or depth of the thin film) and the roughness of the thin film.

**Figure 9:**
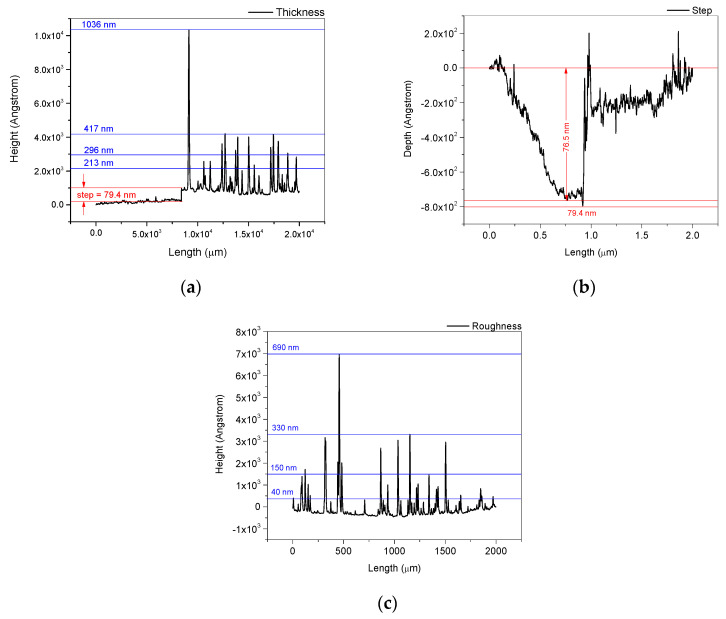
The analysis of the thickness (**a**) of the thin film obtained by DPL method and the thin film thickness components: step (**b**) and roughness (**c**).

The profilometric measurements performed on the side edge of the thin film area and an area inside the thin film showed a step of 76.5–79.4 nm (Figure 9a,b) and a roughness with peaks between 40 nm and 690 nm (Figure 9c). Thus, according to the results presented in Figure 9a, a thickness of 417 nm can be attributed to the thin film. Peaks up to 1 micrometer or higher (Figure 9a) are also present on the surface of the thin layer. The thickness of the thin film is highly influenced by roughness, which is a characteristic specific to the thin layers obtained by pulsed laser deposition. The roughness of the thin layer is consistent with the morphological structures observed in the images obtained in the SEM analysis (Figure 5e–h).

The thickness of the thin layer is influenced by the number of pulses represented by the deposition time, as well as by other parameters. The distance between the target and the deposition support is also one of the parameters that influence the thickness as is the laser energy through the induced plasma threshold. Depending on the purpose of the thin film, roughness is considered to be a suitable feature. Chitosan thin film roughness represents an advantage when good absorption is required due to the increased sorption surface. This feature recommends the chitosan thin films for the fabrication of hemostatic patches as well as matrices in the TDD systems (transdermal drug delivery systems).

It was also demonstrated with the study presented herein on *Saccharomyces cerevisiae* the property of thin layers of chitosan deposited by the DPL method to isolate biological materials from the environment, which leads to good preservation at the cellular level. By miniaturizing pulsed laser deposition systems, it will be possible to “encapsulate” biological cells as well as other materials collected from hostile environments, including during space missions. These materials could be transported in the DPL chitosan-encapsulated form and later studied.

The DPL method that is presented in this work can be used for the fabrication of the matrix for conducting thin films of polymers. This novel method can be extended to produce thin films from different polysaccharides.

## 4. Conclusions

Dual-pulsed laser (DPL) mode used for the ablation of oyster shell material and deposition of thin films proved that two laser beams forming an angle between them, which was 45° in this particular case, and which are incident on the material surface under an angle of 45° produces different effects compared to those obtained with a single-pulsed laser (SPL) whose beam is also incident at an angle of 45° to the surface of the target. The behavior of chitosan deposition during the DPL regime leads to the idea that crystalline structures could be controlled with this technique.

Effects of the thin layers of chitosan obtained by the DPL method when deposited on *Saccharomyces cerevisiae*, which consist of the preservation and/or restoration of biological cells, were also highlighted.

The results of the numerical simulations in COMSOL and Gaussian 6 software provided information in order to assist and complete the spectral and microscopic analyses of the resulting experimental materials.

## Figures and Tables

**Table 1:** EDX results on elemental analysis.

Samples	ELEMENTS											
	Atomic % (Number of Atoms of a Species out of 100 Atoms of All Detected Species)											
	C	O	Na	Ca	Mg	Al	Si	Fe	P	K	N	S
OS target area1	22.88	66.42	2.88	7.82	-	-	-	-	-	-	-	-
OS target area2	24.46	63.55	3.04	8.95	-	-	-	-	-	-	-	-
**Average OS target**	**23.67**	**64.99**	**2.96**	**8.39**	**-**	**-**	**-**	**-**	**-**	**-**	**-**	**-**
OS glass area1	17.13	61.43	10.04	0.31	2.19	0.47	8.42	-	-	-	-	-
OS glass area2	6.17	70.50	12.63	0.14	2.44	-	8.12	-	-	-	-	-
**Average OS glass**	**11.65**	**65.97**	**11.34**	**0.23**	**2.32**	**0.24**	**8.27**	**-**	**-**	**-**	**-**	**-**
OS hemp area1	58.13	41.75	-	0.05	-	-	-	0.07	-	-	-	-
OS hemp area2	63.23	36.57	-	0.07	-	-	-	0.13	-	-	-	-
**Average OS hemp**	**60.68**	**39.16**	**-**	**0.06**	**-**	**-**	**-**	**0.10**	**-**	**-**	**-**	**-**
OSY glass area1	59.57	39.61	-	0.15	0.67	-	-	-	-	-	-	-
OSY glass area2	59.88	39.66	-	0.11	-	-	-	-	0.19	0.16	-	-
**Average OSY glass**	**59.73**	**39.64**	**-**	**0.13**	**0.34**	**-**	**-**	**-**	**0.10**	**0.08**	**-**	**-**
OSY hemp area1	51.95	35.02	-	0.12	-	-	-	-	0.08	0.07	12.76	0.02
OSY hemp area2	58.87	27.97	0.64	0.14	0.20	-	-	-	0.23	0.19	11.70	0.06
**Average OSY hemp**	**58.00**	**36.38**	**0.64**	**0.13**	**0.44**	**-**	**-**	**-**	**0.15**	**0.13**	**12.23**	**0.04**
Y-support AREA 1	48.83	34.49	-	-	-	0.14	-	-	0.18	0.17	16.15	0.04
Y-support AREA 2	50.65	32.11	-	-	-	-	-	-	0.24	0.24	16.68	0.08
**Average Y-support**	**49.74**	**33.30**	**-**	**-**	**-**	**0.14**	**-**	**-**	**0.21**	**0.21**	**16.42**	**0.06**

**Table 2:** FTIR analyzed spectra functional groups.

Functional Groups Vibrations Wavenumber (cm^−1^)								Observations Based on References [1,23,24,38,39,40] and on the Gaussian 6 Simulated IR Spectra
OS Target	HMP	Y-Dry	Y-Support	OS-GLSS	OS-Y-GLSS	OS-HMP	OS-Y- HMP	
-	-	-	-	3896	-	-	3842	OH free stretching 3941, 3815 cm^−1^ specific to chitin and chitosan as per Gaussian simulation, assigned to OH free stretching
3749	-	3738	3738	3749	3740	-	-	OH free stretching Si-OH, Al-OH, H-OH 3716 cm^−1^ specific to chitin and chitosan as per Gaussian simulation, assigned to OH free stretching
-	-	-	3565	-	-	-	-	O-H free and H-bonded stretching, NH free and H-bonded stretching 3518 and 3507 cm^−1^ specific to chitin and chitosan as per Gaussian simulation, assigned to OH free stretching
3437	3482	3439	3469	3437	3437	3475	-	O-H free and H-bonded stretching, NH free and H-bonded stretching
-	-	-	3372	-	-	-	3369	NH free and H-bonded stretching
-	-	3273	3296, 3257	-	-	-	-	O-H free and H-bonded stretching, NH free and H-bonded stretching 3255 and 3205 cm^−1^ specific to chitin and chitosan as per Gaussian simulation, assigned to NH free stretching
-	-	-	3084	-	3054	-	-	Aromatic C−H stretching
2928	2964 2909	2928, 2923	2958, 2923,	-	-	2914	2925	Aliphatic C−H stretching
2838	-	2851	2851	-	2890	-	2858	C−H stretching, cyclohexanes H−C(−N) stretching in amines 2844 and 2719 cm^−1^ specific to chitin and chitosan as per Gaussian simulation, assigned to CH alipjhatic stretching
2510	-	-	-	-	-	-	-	Chelates in CaCO_3_ 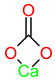
2337	-	2360	2360, 2332	-	-	-	-	Adsorbed gas phase; CO_2_ molecule adsorbed 2318 cm^−1^ specific to chitosan as per Gaussian simulation, assigned to C−N in primary amines
-	2167	-	-	-	-	-	-	Adsorbed CO (usually on metal oxides or generally metals ionic state) C≡N stretching 2114 cm^−1^ specific to chitosan as per Gaussian simulation, assigned to C−N in primary amines
-	-	-	-	-	-	-	2091	C≡C stretching monosubstituted CH aromatic bending
1793	1784	-	-	-	-	-	-	C=O stretching, also in calcium carbonate CaCO_3_ 1750 and 1759 cm^−1^ specific to chitin and chitosan as per Gaussian simulation, assigned to NH bending
1640	1680	1654	1654	1640	1642	-	1648	C=O stretching in primary amides−NHC=O NH bending in primary amines C=C stretching CH aromatic bending 1667 cm^−1^ specific to chitosan as per Gaussian simulation, assigned to O−C and NH bending
1533	-	1545	1545	-	1549	-	1543	NH bending vibration in amines 1533 cm^−1^ specific to chitin and chitosan as per Gaussian simulation, assigned to NH bending
1471	1473	1455	1459	1426	-	-	1460; 1405	−COOH (carboxyl) $-(CO{O)}^{-}$ (carboxylate) bending vibrations $\mathrm{symmetric}\;\mathrm{stretch}\;\mathrm{of}\;\mathrm{carbonate}\;\mathrm{ions}\;CO_{3}^{2-}$ Lattice vibration CH_3_ asymmetric; acetyl group $CH_{3}-C=O$ 1450 cm^−1^ specific to chitin and chitosan as per Gaussian simulation, assigned to OH bending
-	1377	-	-	-	-	1369	-	OH alcoholic bending Corresponds to: 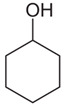 1394 cm^−1^ specific to chitin and chitosan as per Gaussian simulation, assigned to OH bending
-	-	1237	-	-	1229	-	-	1290 cm^−1^ specific to chitin and chitosan as per Gaussian simulation, assigned to skeletal vibrations
-	1162; 1106	-	-	-	-	1155	-	−C−O−C−stretching symmetric vibrations 1100–1108 cm^−1^ specific to chitin and chitosan as per Gaussian sim-ulation, assigned to the bridge C−O−C symmetric vibrations
1082	1045	-	-	1067; 1028;	1050	1062	1062	C-O stretching ${\mathrm{CH}}_{2}-$OH; CH-OH stretching C−O−C stretching asymmetric C-O-O stretching $\mathrm{carbonate}\;\mathrm{ions}\;CO_{3}^{2-}$ symmetric stretching skeletal vibrations due to C−O stretching; 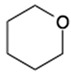 oxane (oxacyclohexane) in glucosamine ring
-	-	986	975	-	988	-	-	−COOH (carboxyl) $-(CO{O)}^{-}$ (carboxylate) bending vibrations; ~925 acetates (acetates and acetic acid resulted from chitin deacetylation) 994 cm^−1^ specific to chitin and chi-tosan as per Gaussian simulation, assigned to the bridge C−O−C asymmetric vibrations
861	858	-	817	877	891	891	-	$\mathrm{Carbonate}\;\mathrm{ions}\;CO_{3}^{2-}$ out-of-plane bending modes 937 cm^−1^ specific to calcium car-bonate, chitin and chitosan as per Gaussian simulation, assigned to the bridge C−O bending vibrations
771	-	-	-	771	-	-	-	CH_2_ bending 771 cm^−1^ specific to calcium carbonate as per Gaussian simulation, assigned to the C=O bending vibrations
-	-	-	-	713	-	-	-	C=O out-of-plane bending modes 713 cm^−1^ specific tas per Gaussian simulation, assigned to C=O out-of-plane bendings in carbonate ions
-	-	-	673	-	-	-	-	CO_2_ molecule adsorbed

## Data Availability

Not applicable.
